# Exploring MR regression patterns in rectal cancer during neoadjuvant radiochemotherapy with daily T2- and diffusion-weighted MRI

**DOI:** 10.1186/s13014-020-01613-4

**Published:** 2020-07-11

**Authors:** T. Bostel, C. Dreher, D. Wollschläger, A. Mayer, F. König, S. Bickelhaupt, H. P. Schlemmer, P. E. Huber, F. Sterzing, P. Bäumer, J. Debus, N. H. Nicolay

**Affiliations:** 1 Clinical Cooperation Unit Radiation Oncology, German Cancer Research Center (DKFZ), Im Neuenheimer Feld 280, 69120 Heidelberg, Germany; 2 Department of Radiation Oncology, University Medical Center Mainz, Langenbeckstrasse 1, 55131 Mainz, Germany; 3 Division of Radiology, German Cancer Research Center (DKFZ), Im Neuenheimer Feld 280, 69120 Heidelberg, Germany; 4 Department of Radiation Oncology, University Hospital Mannheim, Medical Faculty Mannheim, Heidelberg University, Theodor Kutzer-Ufer 1-3, 68167 Mannheim, Germany; 5 Institute of Medical Biostatistics, Epidemiology and Informatics (IMBEI), University Medical Center Mainz, Obere Zahlbacher Strasse 69, 55131 Mainz, Germany; 6 Division of Medical Imaging and Radiology – Cancer Prevention, German Cancer Research Center (DKFZ), Im Neuenheimer Feld 280, 69120 Heidelberg, Germany; 7 Institute of Radiology, Friedrich-Alexander-University Erlangen-Nürnberg, Maximiliansplatz 2, 91054 Erlangen, Germany; 8 Department of Radiation Oncology, University Hospital of Heidelberg, Im Neuenheimer Feld 400, 69120 Heidelberg, Germany; 9 Radiation Oncology, Kempten Clinic, Robert-Weixler-Strasse 50, 87439 Kempten, Germany; 10 dia.log, Altoetting Center for Radiology, Vinzenz-von-Paul-Strasse 10, 84503 Altoetting, Germany; 11 Department of Radiation Oncology, University of Freiburg Medical Center, Robert-Koch-Strasse 3, 79106 Freiburg, Germany

**Keywords:** MRI, Rectal cancer, Chemoradiotherapy, Oncologic imaging, Pathological response, IGRT

## Abstract

**Background:**

To date, only limited magnetic resonance imaging (MRI) data are available concerning tumor regression during neoadjuvant radiochemotherapy (RCT) of rectal cancer patients, which is a prerequisite for adaptive radiotherapy (RT) concepts. This exploratory study prospectively evaluated daily fractional MRI during neoadjuvant treatment to analyze the predictive value of MR biomarkers for treatment response.

**Methods:**

Locally advanced rectal cancer patients were examined with daily MRI during neoadjuvant RCT. Contouring of the tumor volume was performed for each MRI scan by using T2- and diffusion-weighted-imaging (DWI)-sequences. The daily apparent-diffusion coefficient (ADC) was calculated. Volumetric and functional tumor changes during RCT were analyzed and correlated with the pathological response after surgical resection.

**Results:**

In total, 171 MRI scans of eight patients were analyzed regarding anatomical and functional dynamics during RCT. Pathological complete response (pCR) could be achieved in four patients, and four patients had a pathological partial response (pPR) following neoadjuvant treatment. T2- and DWI-based volumetry proved to be statistically significant in terms of therapeutic response, and volumetric thresholds at week two and week four during RCT were defined for the prediction of pCR. In contrast, the average tumor ADC values widely overlapped between both response groups during RCT and appeared inadequate to predict treatment response in our patient cohort.

**Conclusion:**

This prospective exploratory study supports the hypothesis that MRI may be able to predict pCR of rectal cancers early during neoadjuvant RCT. Our data therefore provide a useful template to tailor future MR-guided adaptive treatment concepts.

## Background

Colorectal cancer constitutes the third most common malignant tumor disease with an estimated global incidence of more than one million people per year [1]. The highest annual incidence rates of colorectal cancer are recorded in the developed countries affecting more than 40 per 100.000 people [1]. In the last decades, the cancer-specific mortality rates of rectal cancer have declined significantly owing to considerable advances in treatment as well as improved diagnostic techniques and extended screening measures [2, 3].

Radical resection of the rectum remains the mainstay of curative treatment for rectal cancer; but for locally advanced disease, multimodal therapeutic approaches including radiotherapy (RT) have resulted in significantly improved local control, but no overall survival benefit compared to surgery alone [4, 5]. Neoadjuvant radiochemotherapy (RCT) followed by surgery has emerged as a standard of care for patients with locally advanced rectal cancers, leading to pathologically complete response (pCR) rates of 11–31% [6–8]. Complete responders after neoadjuvant treatment have a favourable outcome with a 5-year disease-free survival of about 83%, making tailored and potentially organ-preserving treatments relevant for these patients in order to reduce therapy-related morbidity and hence improve long-term quality of life [9–11]. In turn, non-responders need to be identified as early as possible, as these patients may benefit from modified and potentially more aggressive treatment concepts and may not be suitable candidates for organ preservation strategies [12].

Consequently, an early prediction of tumor response to neoadjuvant treatment is of special interest. Its assessment may require the monitoring of predictive molecular biomarkers as well as advanced imaging during the course of RCT, although suitable strategies are lacking to date. In recent years, magnetic resonance imaging (MRI) has emerged as the most promising imaging procedure for the prediction of treatment response during and after neoadjuvant RCT [13, 14]. However, available studies are difficult to compare due to inconsistent imaging time points and assessment of different MRI parameters [15–18].

To the best of our knowledge, no data have been published concerning daily anatomical and functional changes of MRI during RCT in rectal cancer. In recent years, our group demonstrated the safety and feasibility of a shuttle-based off-line approach for realizing daily MR-guided radiotherapy [19, 20]. This analysis aimed to measure daily evolutions in tumor volume and apparent diffusion coefficient (ADC), and correlate these findings to the final pathological response outcome to gain new insights into detailed MR regression patterns during neoadjuvant RCT.

## Material and methods

### Patients

Our manuscript analyzed data from a prospective trial investigating daily MRI in treatment position as a means of off-line MR-guided radiotherapy [19, 20]. In this study, 8 patients with locally advanced rectal adenocarcinoma were enrolled between October 2013 and June 2017. All patients completed neoadjuvant RCT. Pathological complete response (pCR; Dworak regression grade 4) could be achieved in 4 patients, while the rest of the patients had a pathological partial response (pPR; Dworak regression grade 1–3) to neoadjuvant treatment [21]. Detailed patient characteristics are provided in Table 1. The trial was carried out in accordance with the Declaration of Helsinki (7th revision) and was approved by the independent ethics commission of the Medical Faculty of the University of Heidelberg (S-144/2013). All patients provided written informed consent prior to inclusion in this trial.

**Table 1:** Patients’ characteristics

Characteristics	Value	Percent
Age (y)		
- Median	63.5	
- Range	51–72	
Gender		
-Female	5	62.5
-Male	3	37.5
Clinical stage before treatment (n)		
-cT2 N1	1	12.5
-cT3 N0–2	7	87.5
Pathological stage (n)		
-ypT0 N0 (pCR)	4	50.0
-ypT2 N0–1	2	25.0
-ypT3 N0–2	2	25.0
Dworak tumor regression grade (TRG)		
-Dominant tumor mass with obvious fibrosis (TRG 1 = minimal regression)	2	25.0
-Dominantly fibrotic changes with few tumor cells or groups (TRG 2 = moderate regression)	1	12.5
-Very few tumor cells (TRG 3 = near pCR)	1	12.5
-No tumor cells (TRG 4 = pCR)	4	50.0
Initial tumor volume (ml)		
-Median	33.5	
-Range	14–95	
Total treatment time (d)		
-Median	40	
-Range	37–43	

Abbreviations: *y* years, *n* number, *pCR* pathological complete response, *TRG* tumor regression grade, *ml* milliliters, *d* days

### Patient immobilization and treatment planning

All patients received daily short MR scans in treatment position prior to irradiation with the same immobilization equipment used for RT to image the morphological and functional changes of the tumorous tissue during the treatment. The transfer of patients between the MR device and the linear accelerator was realized with a shuttle system (Zephyr system, Diacor, Salt Lake City, USA) [19].

Treatment planning was performed using the RayStation™ treatment planning system (RaySearch, Stockholm, Sweden). Gross tumor volume (GTV) was defined as the morphological tumor volume, visible on post-contrast computed tomography and co-registered MRI scans. The clinical target volume (CTV) was defined as proposed by Valentini et al. [22]. The planning target volume (PTV) was defined as the CTV with addition of 5 mm in the horizontal and the cranio-caudal plane.

All patients received 50.4 Gy in 28 fractions and concurrent chemotherapy with 5-fluoruracil (300 mg/m^2^ body surface area daily, administered intravenously via a port catheter system).

Inverse treatment planning was used for intensity-modulated RT. Treatment was carried out using a 6 MV linear accelerator (Siemens Artiste, Erlangen, Germany). Prior to each treatment fraction, position verification imaging was performed using KV cone-beam CT scans.

### MR imaging

Daily MRI examinations were performed for all patients in treatment position immediately before treatment using a 1.5 T MRI scanner (Magnetom Symphony, Siemens Healthcare, Erlangen, Germany) [14]. The MRI protocol included a high-resolution coronal T2-space sequence (TR = 2 s; TE = 125 ms; voxel size = 1.2 × 1.0 × 1.12mm^3^; acquisition matrix 448x288x314), a transverse T1-vibe sequence (TR = 10 ms, TE = 5 ms, voxel size = 2.0 × 2.0 × 2.0mm^3^; acquisition matrix = 228 × 287 × 378) and transverse DWI. For tumor contouring, transverse and sagittal images were reconstructed from the coronal T2-space sequence. DWI was performed in free breathing and in case of baseline MRI prior to administration of contrast agent. The following parameters were used for DWI acquisition: TR = 7.54 s; TE = 96 ms; FOV read/phase 280 mm/76.5%; acquisition matrix 102/0/0/78; Voxel size 2.7 × 2.7 × 3.2 mm; slice thickness 3.2 mm; bandwidth 1442 Hz/Px; b-values 0 and 1000s/mm^2^, gradient mode 3-scan trace; 50 slices in 1 step; 6 min. ADC maps were calculated by a monoexponential fitting model. For a total of 171 of 224 treatment fractions (76%), MRI scans could be conducted and were analyzed regarding anatomical and functional dynamics during RCT.

### Data analysis

GTV was measured using T2-weighted and diffusion-weighted images (DWI). The DWI ROIs were semi-automatically segmented at the setting of b = 1000s/mm^2^ and by the use of the “Medical Imaging Interaction Toolkit” (MITK) transferred to co-registered images at the setting of b = 0 s/mm^2^ [23]. Co-registered T1 and T2 sequences were used to assist visually locating the morphological lesion. To ensure that only areas with restricted diffusivity were considered for volumetry, a threshold tool of the MITK software was used. Further segmentation was performed on each slice, in all sections and within inner limits to reduce partial volume effects. After semi-automatic generation of the DWI-based tumor volumes, these were checked by the two radiologists and manually corrected if necessary. ADC values in the DWI ROIs were calculated by a monoexponential fitting model, based on a software code developed in MATLAB (MathWorks, Natick, Massachusetts).

Volumetric and functional MR data were defined by the consensus of two diagnostic radiologists. Both radiologists were blinded for the pathological response.

### Statistical analysis

Tumor volume at each radiation fraction of RCT was compared with the corresponding volume at the first and previous treatment fraction. Furthermore, tumor volumes and ADC values at baseline as well as at weeks 2 and 4 after initiation of RT were compared between the response groups (i.e., pathological complete or partial response) using the Wilcoxon-Mann-Whitney test with a significance level of *p* < 0.05. These time points were chosen because they offered complete data. Due to the exploratory nature of the study, no formal adjustment for multiple testing was carried out. Statistical analysis was performed by the R software package, version 3.5.1 (R Core Team 2018, Vienna, Austria).

## Results

### Volumetric analysis

#### T2-based volumetry

**Fig. 1:**
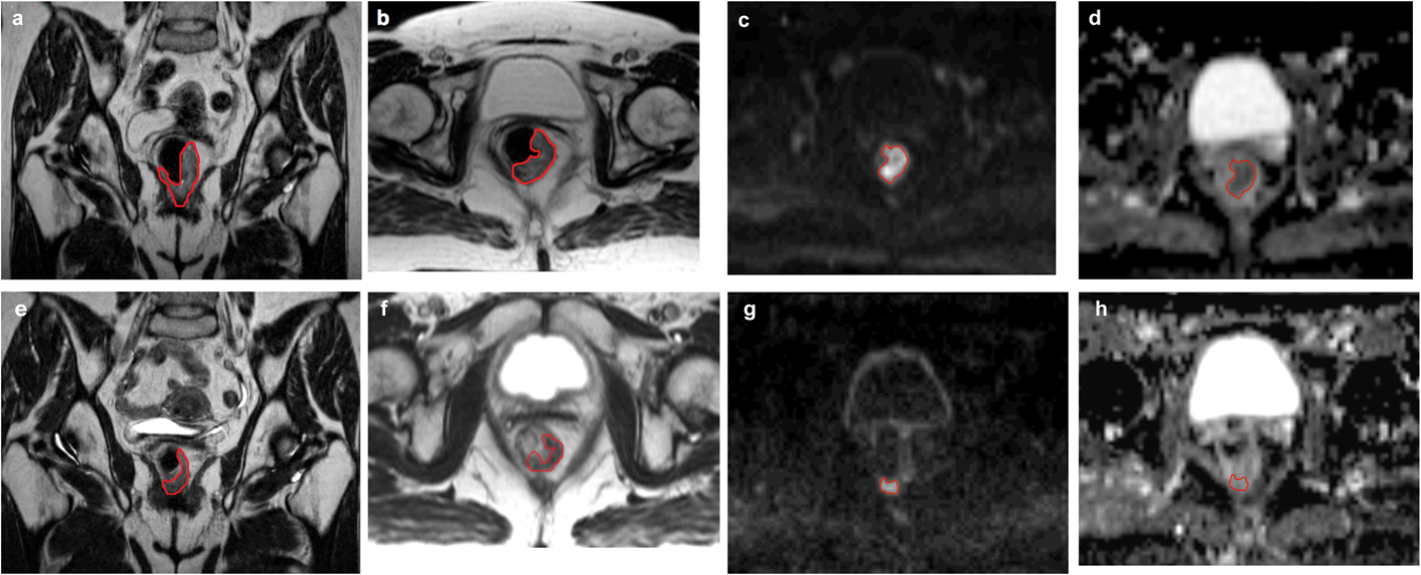
Example of tumor regression in a single patient between the first and last fraction of neoadjuvant treatment. Distinct decrease in gross tumor volume (GTV; red outlined) is evident (**a + b**: coronal and transverse T2-weighted images at baseline, **e + f**: coronal and transverse T2-weighted images at the end of neoadjuvant RCT). The diffusion-restricted area within the GTV (red) also declines until end of RCT, indicating the treatment-related cell depletion (**c + d**: transverse DWI sequence obtained with a b-value of 1000 s/mm^2^ and corresponding ADC-map at baseline, **g + h:** transverse DWI sequence (b-value = 1000) and ADC-map at the end of neoadjuvant RCT; the segmented GTV (red) on the ADC-maps is given to highlight the tumor, however segmentation for the ADC calculation has been performed on high b-value images

**Fig. 2:**
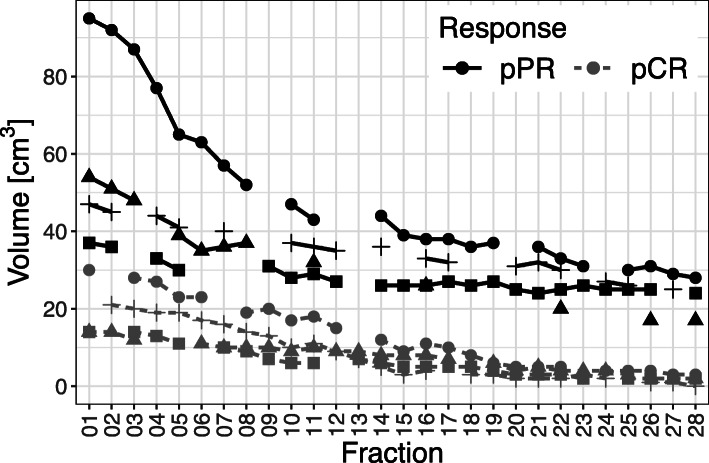
Separate presentation of gross tumor volume dynamics for each patient during neoadjuvant RCT (based on T2w sequence). Between the complete and partial pathological responders (pCR and pPR) distinct differences of absolute tumor volumes were evident at baseline as well as over the whole course of RCT

**Fig. 3:**
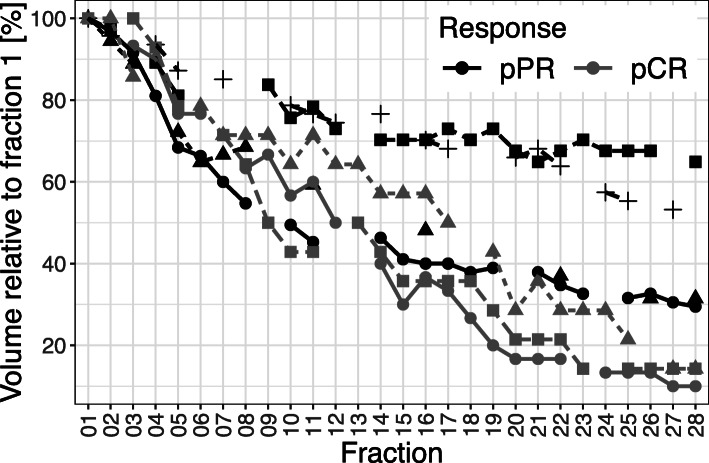
Separate presentation of gross tumor volume changes during RCT relative to the first fraction of treatment for each patient (based on T2w sequence). While in the first 2 weeks all patients exhibited substantial tumor shrinkage, distinct differences were evident between pathological complete and partial responders (pCR and pPR) in the further course of RCT

**Fig. 4:**
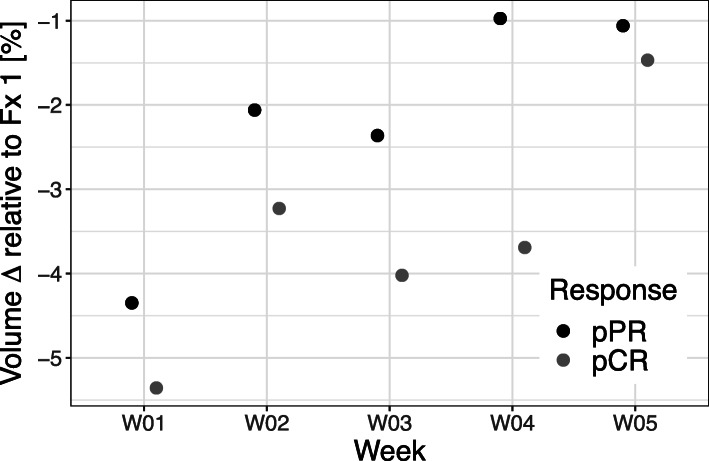
The points in the figure represent average daily volume changes per week relative to the first fraction of neoadjuvant RCT (baseline) for complete and partial pathological responders (pCR and pPR) separately. Complete and partial pathological responders (pCR and pPR) differed substantially, particularly in the first 4 weeks of treatment

**Fig. 5:**
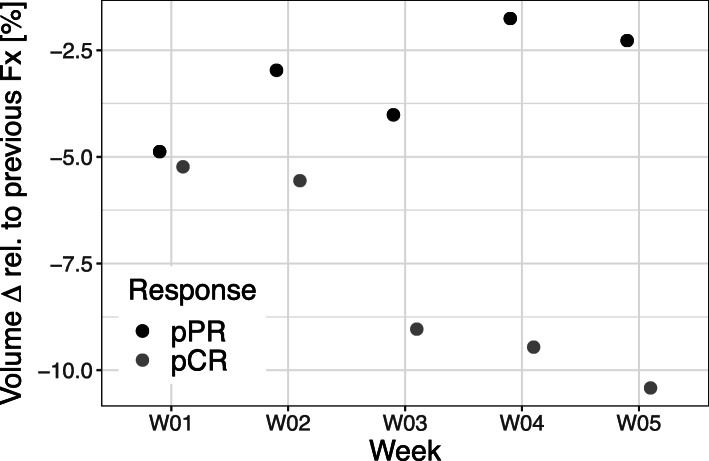
The points in the figure represent average daily volume changes per week relative to the previous fraction of neoadjuvant RCT for complete and partial pathological responders (pCR and pPR) separately. While pathological complete responders (pCR) exhibited steadily increasing tumor shrinkage rates from week to week, pathological partial responders (pPR) showed largely stable or even declining tumor shrinkage rates during RCT

All patients exhibited a distinct volume reduction of the tumor during the course of RCT (see Figs. 1 and 2) with most pronounced changes in patients reaching a pCR (see Table 2): From baseline to the end of neoadjuvant treatment, the average T2-based tumor volume decreased from 39 cm^3^ (range 14–95 cm^3^) to 10.9 cm^3^ (range 0–28 cm^3^) Patients with a pCR had a significant lower absolute T2-tumor volume at the beginning of RCT as well as after 2 and 4 weeks of treatment (fraction 11 and fraction 22) than those patients with a pPR (see Fig. 2 and Table 2). The T2-based tumor volumes of patients with pCR and pPR averaged 19.3 cm^3^ (range 14–30 cm^3^) and 58.2 cm^3^ (range 37–95 cm^3^) prior to RCT (*p* = 0.03), 11.3 cm^3^ (range 6–18 cm^3^) and 35.0 cm^3^ (range 29–43 cm^3^) at 2 weeks after the initiation of RCT (*p* < 0.01), and 3.5 cm^3^ (range 2–5 cm^3^) and 27.0 cm^3^ (range 20–33 cm^3^) at week 4 during RCT (*p* < 0.01), respectively. Until the end of neoadjuvant treatment, the mean relative tumor shrinkage rate was 76.7% in the overall study population, 87.1% in the pCR group and 58.1% in the pPR group as compared to the baseline tumor volume. The fastest tumor shrinkage rate relative to the baseline volume was observed during the first 2 weeks of treatment independently from the pathological treatment response. However, in the following weeks distinct differences of tumor shrinkage were evident between both response groups (see Figs. 1 and 3). In the overall study population and the subgroups of patients with pCR and pPR, average tumor volume decreased by 2.7, 3.1 and 2.1%, respectively, per radiation fraction compared to baseline volume (please see Fig. 4 and Table 2 for detailed description of daily rectal tumor volume changes). In the overall study population and the subgroups of patients achieving pCR and pPR, tumor volume declined on average by 7.0, 9.0 and 4.2% from one fraction to the next. The biggest differences of daily tumor shrinkage between both response groups were observed in the last 3 weeks of RCT; in the pCR group, average daily tumor shrinkage was steadily increasing during RCT, while in the pPR group the tumor shrinkage rates were considerably lower and decreasing until the end of treatment (see Fig. 5 and Table 2).

**Table 2:** Comparison of T2-based tumor volumetry between response groups

	T2-based tumor volumetry (ml)											
	pCR						pPR					
	Mean	Min	Max	Mean rel. to BL (%)	Mean rel. to PM (ml)	n	Mean	Min	Max	Mean rel. to BL (%)	Mean rel. to PM (%)	n
**Fx 1**	19.3	14	30	100		3	58.2	37	95	100		4
**Fx 2**	17.5	14	21	100	0	2	56.0	36	92	96.1	4	4
**Fx 3**	18.5	12	28	93.0	6.5	4	67.5	48	87	90.2	5.5	2
**Fx 4**	19.7	13	27	91.4	5.3	3	51.3	33	77	88.0	7	3
**Fx 5**	17.7	11	23	77.6	10	3	43.8	30	65	77.2	12.8	4
**Fx 6**	17.0	11	23	77.6	6.3	3	49.0	35	63	65.6	6.5	2
**Fx 7**	12.0	10	16	71.4	8	3	44.3	36	57	70.6	4	3
**Fx 8**	13.0	9	19	66.3	9.8	4	44.5	37	52	61.6	4.5	2
**Fx 9**	12.5	7	20	62.7	7.3	4	31.0	31	31	83.8	0	1
**Fx 10**	10.5	6	17	54.6	15.5	4	37.3	28	47	68.0	9	3
**Fx 11**	11.3	6	18	58.1	0	4	35.0	29	43	64.9	6.5	4
**Fx 12**	11.0	9	15	57.1	15	3	31.0	27	35	73.7	5	2
**Fx 13**	7.7	7	9	57.1	7.3	3	NA	–	–	NA	NA	0
**Fx 14**	7.8	5	12	46.7	18.5	4	35.3	26	44	64.4	1.3	3
**Fx 15**	6.3	3	9	41.0	20.5	4	32.5	26	39	55.7	5.5	2
**Fx 16**	7.0	4	11	43.2	0	4	30.8	26	38	57.2	7.5	4
**Fx 17**	7.3	5	10	39.7	7	3	32.3	27	38	60.4	1	3
**Fx 18**	5.3	3	8	31.2	15	3	31.0	26	36	54.1	4.5	2
**Fx 19**	4.8	3	6	30.5	14.8	4	32.0	27	37	56.0	0	2
**Fx 20**	3.5	2	5	22.2	27	4	28.0	25	31	66.8	5	2
**Fx 21**	3.8	2	5	24.6	0	4	30.7	24	36	57.0	2.3	3
**Fx 22**	3.5	2	5	22.2	5	4	27.0	20	33	50.8	9.3	4
**Fx 23**	3.0	2	4	21.4	11	3	28.5	26	31	51.5	3	2
**Fx 24**	3.3	2	4	20.9	17.8	3	26.0	25	27	62.5	7	2
**Fx 25**	3.0	2	4	16.4	8.3	3	27.0	25	30	51.5	2.3	3
**Fx 26**	2.3	1	4	13.8	16.7	3	24.3	17	31	43.9	5	3
**Fx 27**	2.0	1	3	12.9	14.5	4	27.0	25	29	41.9	5	2
**Fx 28**	1.8	0	3	12.9	25	4	23.0	17	28	41.9	2.3	3

*Abbreviations: ml* milliliters, *pCR* pathological complete response, *pPR* pathological partial response, *rel.* relative, *BL* baseline, *PM* previous measurement, *Min* minimum, *Max* maximum, *Fx* fraction, *NA* not analyzable, i.e. no values existent

#### DWI-based volumetry

In general, the tumor volumes assessed using DWI (b = 1000s/mm^2^) were lower than those assessed by T2-based imaging (see Table 3). At baseline, no significant difference in average DWI-based tumor volumes between the pCR and pPR groups was evident (7.8 vs. 23.4 cm^3^, *p* = 0.10). In accordance with the T2-based volume assessment, there were distinct volume changes during RCT (see Table 3). From baseline to the end of neoadjuvant treatment, the average DWI-based tumor volume of the overall study population decreased from 16.7 cm^3^ (range 5.4–38.3 cm^3^) to 5.0 cm^3^ (range 0–28 cm^3^) After 2 weeks of neoadjuvant treatment, DWI-based tumor volumes were lower in the pCR group compared to the pPR group (pCR vs. pPR: average volume 2.0 cm^3^ vs. 11.9 cm^3^, *p* = 0.06). After 4 weeks of RCT, absolute DWI-based tumor volumes of pathologically complete responders were significantly lower than for partial responders (2.9 cm^3^ vs. 7.9 cm^3^, *p* = 0.03).

**Table 3:** Comparison of DWI-based tumor volumetry between response groups

	DWI-based tumor volumetry (ml)									
	pCR					pPR				
	Mean	Min	Max	Mean rel. to BL (%)	n	Mean	Min	Max	Mean rel. to BL (%)	n
**Fx 1**	7.8	5.4	11.6	100	3	23.4	5.9	38.3	100	4
**Fx 2**	5.5	5.3	5.7	91.2	2	17.3	6.4	31.5	78.4	4
**Fx 3**	7.6	5.6	10.4	107.7	4	32.6	26.0	39.2	91.0	2
**Fx 4**	4.2	3.0	5.2	46.5	3	13.2	6.1	26.6	74.1	3
**Fx 5**	4.7	2.7	6.2	51.8	3	11.3	3.5	25.2	52.2	4
**Fx 6**	4.4	2.9	5.8	58.7	3	13.8	9.4	18.2	38.2	2
**Fx 7**	5.2	3.2	6.7	75.5	3	11.5	5.7	18.4	38.0	3
**Fx 8**	4.7	2.3	7.1	65.8	4	18.5	16.1	20.8	51.9	2
**Fx 9**	4.0	1.8	6.7	60.3	4	7.6	7.6	7.6	128.5	1
**Fx 10**	4.2	2.1	6.7	66.2	4	14.4	5.5	30.0	72.8	3
**Fx 11**	4.6	2.5	7.2	70.5	4	11.9	4.5	21.3	54.9	4
**Fx 12**	3.8	1.8	5.6	52.3	3	6.7	6.6	6.8	75.9	2
**Fx 13**	3.9	2.6	6.5	76.6	3	NA	–	–	NA	0
**Fx 14**	4.5	1.1	7.3	79.1	4	13.7	4.4	19.0	78.0	3
**Fx 15**	3.6	1.7	5.7	58.1	4	14.4	4.3	24.6	68.1	2
**Fx 16**	3.5	0.9	6.7	53.3	4	9.7	3.7	14.4	46.7	4
**Fx 17**	3.7	1.2	7.7	57.2	3	7.8	2.4	13.3	40.5	3
**Fx 18**	2.5	1.2	3.4	31.7	3	9.3	3.0	15.6	45.3	2
**Fx 19**	3.4	1.5	7.2	57.6	4	8.0	3.3	12.7	44.4	2
**Fx 20**	3.7	1.2	6.0	65.4	4	5.5	5.4	5.5	62.3	2
**Fx 21**	3.4	1.0	6.7	54.9	4	5.6	3.5	7.3	39.8	3
**Fx 22**	2.9	1.1	5.3	47.0	4	7.9	5.1	10.0	46.4	4
**Fx 23**	3.3	2.2	4.6	66.3	3	8.1	4.1	12.1	50.1	2
**Fx 24**	3.5	1.6	6.7	60.6	3	4.5	3.3	5.7	44.6	2
**Fx 25**	2.8	1.4	5.1	43.4	3	6.8	3.8	9.8	43.2	3
**Fx 26**	1.6	1.1	2.4	26.8	3	9.1	3.5	12.1	42.1	3
**Fx 27**	2.7	0.7	6.6	52.1	4	10.1	6.1	14.0	36.6	2
**Fx 28**	2.4	0.7	5.8	44.4	4	8.4	4.2	16.2	42.5	3

*Abbreviations: ml* milliliters, *pCR* pathological complete response, *pPR* pathological partial response, *rel.* relative, *BL* baseline, *Min* minimum, *Max* maximum, *Fx* fraction, *NA* not analyzable, i.e. no values existent

### ADC analyses

When comparing patients with pCR and pPR, the mean tumor ADC values at baseline as well as during the course of RCT widely overlapped; showing no statistically significant group difference for any ADC dynamics measures. The baseline average ADC values were comparable in both response groups (pCR: 1.04 × 10^− 3^ mm^2^/s, range 0.92–1.25 × 10^− 3^ mm^2^/s; pPR: 1.04 × 10^− 3^ mm^2^/s, range 0.96–1.10 × 10^− 3^ mm^2^/s; *p* = 0.30); during RCT, the average ADC values at weeks 2 and 4 after initiation of RCT were 1.31 × 10^− 3^ mm^2^/s (range 1.22–1.37 × 10^− 3^ mm^2^/s) and 1.43 × 10^− 3^ mm^2^/s (range 1.38–1.50 × 10^− 3^ mm^2^/s) in the pCR group and 1.33 × 10^− 3^ mm^2^/s (range 1.21–1.46 × 10^− 3^ mm^2^/s) and 1.44 × 10^− 3^ mm^2^/s (range 1.27–1.67 × 10^− 3^ mm^2^/s) in the pPR group (*p* = 0.60 and *p* = 0.70). The detailed course of ADC values during neoadjuvant RCT is summarized in Table 4.

**Table 4:** Comparison of ADC-values between response groups

	ADC values (10^**−3**^ mm^**2**^/s)									
	pCR					pPR				
	Mean	Min	Max	Mean rel. to BL (%)	n	Mean	Min	Max	Mean rel. to BL (%)	n
**Fx 1**	1.04	0.92	1.25	100	3	1.04	0.96	1.10	100	4
**Fx 2**	1.10	0.89	1.32	105.1	2	1.02	0.95	1.08	97.6	4
**Fx 3**	0.98	0.93	1.12	96.2	4	1.04	1.02	1.05	98.5	2
**Fx 4**	0.99	0.90	1.06	104.5	3	1.09	1.00	1.17	105.9	3
**Fx 5**	1.13	0.98	1.34	127.9	3	1.07	1.01	1.19	102.9	4
**Fx 6**	1.17	1.05	1.24	116.0	3	1.12	1.11	1.12	106.0	2
**Fx 7**	1.18	1.05	1.30	106.5	3	1.16	1.09	1.20	108.3	3
**Fx 8**	1.19	1.09	1.29	116.4	4	1.20	1.14	1.27	114.0	2
**Fx 9**	1.24	1.14	1.39	124.6	4	1.30	1.30	1.30	134.9	1
**Fx 10**	1.21	1.13	1.29	119.1	4	1.29	1.20	1.35	126.0	3
**Fx 11**	1.31	1.22	1.37	127.7	4	1.33	1.21	1.46	127.8	4
**Fx 12**	1.31	1.24	1.39	125.6	3	1.26	1.23	1.30	123.4	2
**Fx 13**	1.24	1.13	1.38	118.8	3	NA	–	–	NA	0
**Fx 14**	1.36	1.26	1.44	133.6	4	1.30	1.24	1.39	127.5	3
**Fx 15**	1.35	1.27	1.43	131.4	4	1.43	1.37	1.48	143.9	2
**Fx 16**	1.40	1.29	1.50	137.3	4	1.34	1.23	1.52	128.7	4
**Fx 17**	1.38	1.26	1.55	135.5	3	1.33	1.23	1.46	129.9	3
**Fx 18**	1.45	1.30	1.55	151.9	3	1.34	1.24	1.44	135.1	2
**Fx 19**	1.38	1.32	1.43	133.1	4	1.36	1.28	1.43	137.0	2
**Fx 20**	1.33	0.98	1.50	123.2	4	1.35	1.21	1.48	132.3	2
**Fx 21**	1.52	1.38	1.69	150.0	4	1.31	1.25	1.44	128.6	3
**Fx 22**	1.43	1.36	1.50	138.0	4	1.44	1.27	1.67	138.2	4
**Fx 23**	1.43	1.39	1.49	129.4	3	1.40	1.39	1.40	140.8	2
**Fx 24**	1.47	1.35	1.55	137.8	3	1.37	1.26	1.48	134.5	2
**Fx 25**	1.44	1.38	1.50	140.5	3	1.36	1.26	1.51	133.6	3
**Fx 26**	1.41	1.36	1.49	145.3	3	1.53	1.43	1.65	150.2	3
**Fx 27**	1.51	1.46	1.54	147.1	4	1.35	1.27	1.43	127.3	2
**Fx 28**	1.50	1.38	1.60	143.7	4	1.53	1.45	1.63	150.1	3

*Abbreviations: pCR* pathological complete response, *pPR* pathological partial response, *rel.* relative, *BL* baseline, *Min* minimum, *Max* maximum, *Fx* fraction, *NA* not analyzable, i.e. no values existent

## Discussion

For the first time this exploratory study prospectively evaluated the predictive value of fractional MRI examinations for the early assessment of regression patterns in rectal cancer patients during neoadjuvant RCT.

In our analysis, T2-based tumor volume at baseline as well as at weeks 2 and 4 (fraction 11 and 22) of neoadjuvant RCT significantly correlated with the patients’ pCR rates as assessed post surgery. In this context, a consistent tumor shrinkage relative to the initial tumor volume was observed during RCT, whereby the most considerable changes manifested in the first 2 weeks of treatment. Until the end of RCT, the average T2-tumor volume decreased by 87% in the pCR group and by 58% in pPR patients. When daily rectal tumor volume changes were compared to the volume measured during the previous treatment fraction, average tumor shrinkage rates steadily increased in the pCR group while they decreased in the pPR group. Our data support the hypothesis that a pCR may be predicted early during neoadjuvant RCT based on MRI patterns as tumor volume thresholds of below 25 ml after 2 weeks and 10 ml after 4 weeks correlated with the achievement of a post-RT pCR. However, the proposed threshold values should be considered with caution due to the small number of patients in our analysis, which need to be verified in larger future studies.

Several analyses based on MRI suggested tumor volume reductions of 70–75% from baseline until re-staging prior to surgery to be associated with a pCR [24–26], and a recent observational study with weekly MRI reported similar tumor volume changes as compared to those we found in this analysis, supporting the predictive value of tumor volume regression during RCT [15].

Further published data investigating the predictive power of ADC changes in the context of neoadjuvant treatment showed conflicting results [27–30]. Intven et al. proposed an initial ADC value of 0.97 × 10^− 3^ mm^2^/s and an ADC difference of 41% between pre-RCT and pre-surgery MRI scans as a cut-off for differentiation between good and moderate/poor responders [17]. In contrast, ADC values could not predict pathological treatment response in our analysis as ADC values widely overlapped between complete and non-complete responders during RCT. In this context, the small number of patients in our prospective study has to be considered concerning the interpretation of results as single outliers may already distort the statistical analysis. Nevertheless, our data do not support the suggested predictive value of delta ADC measurements during RCT.

Several studies analyzed a predictive potential of DWI volumetry for the identification of rectal cancer patients with a pCR [30]. One available study proposed that DWI volumetry after RCT offered the best results for the prediction of pCR with a sensitivity of 70% and a specificity of 98% [31]. To the best of our knowledge, no data concerning daily DWI volumetry during RCT have been reported. In our analysis, significant differences of DWI-based tumor volumes between patients with pCR and pPR were observed at week 4 during neoadjuvant RCT. In contrast, neither the baseline DWI volumetry nor the relative volume decreases showed a significant difference between both response groups in our dataset. Our data suggest that pathological response can be predicted by DWI volumetry during neoadjuvant treatment, although our findings have to be corroborated in larger patient cohorts. In addition, this analysis revealed significant differences in T2 and DWI volumetry, which may be explained on the one hand by the difference between tumor volumetry based on morphological MRI sequences as compared to functional DWI and on the other hand by the semi-automatic segmentation approach in case of DWI.

The detailed description of daily volumetric changes in this study aims to serve as a template to design further trials to confirm the role of MRI as a means for early response assessment in order to devise response-specific adaptive treatment strategies. Tumor volume decreases during RCT offer the option to adapt treatment volumes for a better sparing of the surrounding tissues-at-risk with a potential impact on acute and late radiogenic toxicities as well as perioperative morbidity [15]. There is an increasing interest in tailoring neoadjuvant treatments more closely to the extent of tumor regression during RCT based on MRI examinations [32]. Therefore, MR parameters have to be defined for reliable differentiation between good and moderate or poor responders during neoadjuvant treatment [15, 16]. Particularly poor and moderate responders may benefit from dose escalation strategies in order to increase local control and survival rates. However, the implementation of adaptive approaches in the daily routine is difficult for practical reasons. On the other hand, adaptive radiotherapy (ART) including boost strategies might result in higher clinical complete response rates (cCR), which has been suggested to influence progression-free-survival [33]. In recent years, RCT with consecutive cCR or pCR may allow wait-and-watch approaches or local tumor excision strategies, thereby sparing patients mutilating surgeries and improving treatment-related toxicities and quality of life [9, 34, 35]. While standard RCT regimes result in cCR rates of about 17–28%, considerably higher cCR rates up to 70% seem to be achievable by higher-dose RCT approaches [9, 36–39].

This study mainly serves as an accurate template for determining appropriate measurement times for DWI and volumetric monitoring during RCT that may be applicable to novel MRI-guided radiotherapy approaches. This is of special interest for treatment concepts using hybrid MRI-linear accelerators, enabling integrated daily fractional MRI [40–42]. However, it is important to note the different capabilities of diagnostic MRI (as performed in this prospective study) and imaging provided by hybrid devices, especially with regard to DWI requiring b0 homogeneity [43].

Despite the complex and comprehensive MR imaging protocols used here, the small number of patients limits our analysis, and corroboration of our findings in a larger patient cohort is warranted. Furthermore, the sample is not fully representative of the general population, as the pCR rate in our dataset amounted to 50%; nevertheless, as the focus of our analysis was to analyze and report the longitudinal course of MR regression patterns during neoadjuvant treatment in detail, we feel that this deviation of the pCR rates from previous cohorts should not invalidate our data [44, 45]. This analysis does not include inter-observer evaluation, which could be a potentially important factor, since reported interfractional volumetric differences during neoadjuvant treatment could be within the range of expected differences between independent observers. To date, no other data are available reporting daily anatomical and functional MR data for rectal cancer patients undergoing neoadjuvant radiochemotherapy. Therefore, this study provides a unique comprehensive dataset, which can help to gain valuable new insights into detailed MR regression patterns during neoadjuvant treatment of rectal cancers and the trends observed from this data may serve as a useful template for future MR-guided radiotherapy studies.

## Conclusion

This prospective study supports the hypothesis that MRI may be able to predict pCR of rectal cancers early during neoadjuvant RCT. Our data therefore provide a useful template to tailor future MR-guided adaptive treatment concepts.

## Data Availability

The datasets used and/or analysed during the current study are available from the corresponding author on reasonable request.

## Abbreviations

MRI: Magnetic resonance imaging
MR: Magnetic resonance
RCT: Radiochemotherapy
RT: Radiotherapy
ART: Adaptive radiotherapy
DWI: Diffusion-weighted-imaging
ADC: Apparent diffusion coefficient
pCR: Pathological complete response
pPR: Pathological partial response
cCR: Clinical complete response
GTV: Gross tumor volume
CTV: Clinical target volume
PTV: Planning target volume
MV: Megavolt
KV: Kilovolt
T: Tesla
TR: Time of repetition
TE: Time of echo
mm: Millimeter
cm: Centimeter
ms: Milliseconds
s: Seconds
FOV: Field of view
ROI: Region of interest
MITK: Medical Imaging Interaction Toolkit
